# Cholestatic Hepatic Injury Induced by Carbimazole: A Case Report and a Thorough Literature Review

**DOI:** 10.1002/ccr3.70498

**Published:** 2025-06-19

**Authors:** Bassem Al Hariri, Mohammed Omer Elbadawi Elhassan, Hadil Altaj Altrify Alsidig, Muad Abdi Hassan, Abdalrahman Mohammed Mostafa, Memon Noor Illahi

**Affiliations:** ^1^ Department of Medicine Hamad Medical Corporation Doha Qatar; ^2^ College of Medicine Qatar University Doha Qatar; ^3^ College of Medicine Weill Cornell Medicine – Qatar Doha Qatar; ^4^ Medical Education Department Hamad Medical Corporation Doha Qatar

**Keywords:** carbimazole, cholestatic liver injury, drug‐induced liver injury (DILI), hepatotoxicity, hyperthyroidism, thionamide therapy

## Abstract

Carbimazole, a thionamide derivative, is widely used for hyperthyroidism but carries a rare risk of hepatotoxicity, including cholestatic injury. A 42‐year‐old man developed severe cholestatic jaundice 4 weeks after carbimazole initiation. Laboratory findings showed marked hyperbilirubinemia (peak: 650 μmol/L) with elevated ALP (366 U/L). Viral, autoimmune, and biliary causes were excluded. Liver biopsy confirmed cholestasis. Carbimazole was discontinued, corticosteroids initiated, and radioiodine therapy planned. Bilirubin levels gradually improved over 8 weeks. Carbimazole‐induced cholestasis, though rare, necessitates prompt recognition and alternative hyperthyroidism management. Routine liver monitoring is crucial.

**Summary**

- Carbimazole‐induced hepatic injury is an extremely rare adverse event.
- However, this should be considered in patients presenting with jaundice and thyrotoxicosis.
- Prompt discontinuation of the drug and transition to other definitive treatments for hyperthyroidism are mandatory.

## 1 Introduction

Carbimazole, a thionamide derivative, is a cornerstone in the management of hyperthyroidism, particularly in conditions such as Graves' disease. While it is effective in reducing thyroid hormone levels, its use is not without risk. One of the more serious adverse effects associated with carbimazole therapy is hepatotoxicity, which, although rare, poses significant clinical challenges. The incidence of carbimazole‐induced hepatotoxicity is reported to be between 0.1% and 0.2% of patients treated, with manifestations ranging from mild liver enzyme elevations to severe cholestatic jaundice and acute liver failure [1].

The mechanisms underlying carbimazole‐induced liver injury are not fully elucidated, but it is believed to involve an immuno‐allergic response, potentially exacerbated by the drug's metabolic pathways [2]. Histological examinations of liver biopsies from affected patients often reveal portal tract edema and inflammation, indicative of a cholestatic process [3]. Clinicians must remain vigilant for signs of liver dysfunction, particularly in the early weeks of treatment, as the onset of hepatotoxicity can occur relatively quickly, often within the first month of therapy [1, 4].

In the context of hyperthyroidism management, the presence of hepatotoxicity complicates treatment decisions. While some literature suggests switching to alternative antithyroid medications such as propylthiouracil (PTU) may be beneficial, this approach carries its risks, including the potential for cross‐reactivity and similar adverse effects [2, 4]. Therefore, a comprehensive understanding of the risks associated with carbimazole, including the potential for hepatotoxicity, is essential for healthcare providers involved in the management of hyperthyroid patients [2].

This case report aimed to highlight the complexities and clinical implications of carbimazole‐induced hepatotoxicity, providing insights into patient management and the need for careful monitoring during treatment. By presenting a detailed case, we hope to contribute to the existing body of knowledge and enhance awareness of this critical issue among healthcare professionals.

## 2 Case History/Examination

A 42‐year‐old East Asian man presented to the emergency department with sudden‐onset weakness in both upper and lower limbs following an overnight heavy meal. He denied numbness, facial involvement, speech difficulties, visual changes, gastrointestinal symptoms, respiratory issues, or genitourinary complaints. His medical history was unremarkable, and he was not on any medications. He was a nonsmoker and an ex‐alcoholic, having abstained for the past year.

On examination, he was alert, oriented, and comfortable, with no jaundice or pallor. Cardiovascular and respiratory findings were normal, and his abdomen was soft and non‐tender. Neurological examination revealed weakness in all four limbs with intact sensation. Laboratory results showed critically low potassium levels (2.0 mmol/L), suppressed TSH (< 0.01 mIU/L), and elevated FT3 (16.3 pmol/L) and FT4 (44.7 pmol/L). ECG findings were consistent with hypokalemia, showing prominent U waves.

Approximately 4 weeks later, the patient returned to the hospital with a 1‐week history of progressive jaundice, severe pruritus, dark urine, pale stools, and mild epigastric abdominal pain unrelated to food intake. He denied fever, joint pain, nausea, vomiting, or recent illness. Examination revealed deep jaundice and fine tremors without evidence of goiter, organomegaly, or ascites. Laboratory investigations revealed markedly elevated bilirubin levels (total bilirubin 248 μmol/L, direct bilirubin 216 μmol/L), elevated ALP (366 U/L), and mildly elevated ALT (54 U/L) and AST (35 U/L) (Table 1). Hematological parameters were normal.

**TABLE 1:** Results of routine blood tests on initial and subsequent admissions.

Parameter	Normal range	Initial admission	Second admission (jaundice)	Follow‐up/third admission
Potassium (mmol/L)	3.5–5.0	2.0	**3.3 L**	**2.7 L**
TSH (mIU/L)	0.4–4.0	**< 0.01 L**	**< 0.01 L**	**< 0.01 L**
FT3 (pmol/L)	3.7–6.4	**16.3 H**	6.1	**3.2 L**
FT4 (pmol/L)	11.0–23.3	**44.7 H**	**25.5 H**	16.3
Total bilirubin (μmol/L)	< 21	6	**248 H**	**560–650 H**
Direct bilirubin (μmol/L)	< 5	—	**216 H**	**582 H**
ALT (U/L)	0–41	**47 H**	**54 H**	**74 H**
AST (U/L)	0–40	26	35	**57 H**
ALP (U/L)	40–129	**204 H**	**366 H**	**257 H**
Autoimmune markers	Negative	Negative	Negative	—
Thyroid antibodies	Negative	Positive	Positive	—

*Note:* Bold values indicate abnormal values.

Imaging studies, including abdominal ultrasound, showed a normal‐sized liver with smooth echogenicity, a partially contracted gallbladder containing sand‐like stones, and no biliary dilatation or obstruction. MRCP confirmed diffuse adenomyomatosis of the gallbladder without choledocholithiasis or biliary obstruction. Extensive investigations, including a hepatitis panel, HIV test, and autoimmune markers (ANA, ANCA, AMA, and SMA), were negative. Antithyroid peroxidase and antithyroglobulin antibodies were positive.

## 3 Differential Diagnosis, Investigations, and Treatment

During his initial admission for limb weakness, the patient was admitted to the MICU for severe hypokalemia and close monitoring. He was treated with intravenous and oral potassium supplementation, leading to the normalization of potassium levels within 24 h. Further workup confirmed autoimmune hyperthyroidism (TSH receptor antibody positive), and he was started on carbimazole 20 mg BID and atenolol 25 mg daily. His symptoms resolved, and he was discharged 2 days later with endocrine follow‐up planned for hyperthyroidism and newly identified prediabetes.

On his subsequent admission for jaundice and pruritus, carbimazole‐induced hepatotoxicity was suspected, and the drug was immediately discontinued. He was treated with cholestyramine 4 g TID for pruritus, and hepatotoxic medications were avoided. His bilirubin levels stabilized, and he was discharged after 9 days with instructions for close monitoring of liver function.

**FIGURE 1:**
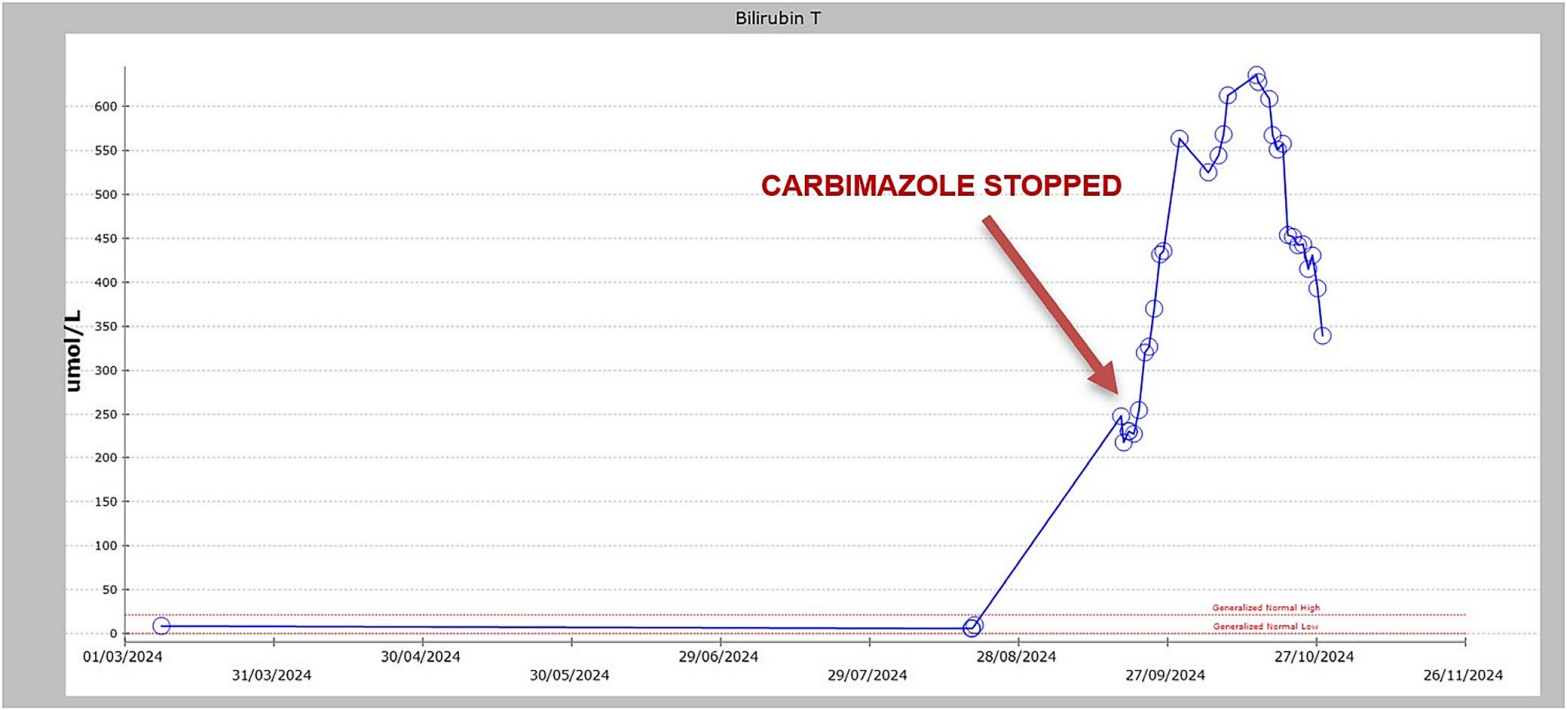
Illustrate the trendline of bilirubin levels after stopping carbimazole. After stopping carbimazole, bilirubin levels remained high for 2–3 weeks.

However, during a follow‐up visit 3 days later, his bilirubin levels were found to have risen significantly to 560 μmol/L. The patient was electively readmitted for further evaluation. Over the following days, his bilirubin levels exceeded 650 μmol/L despite carbimazole discontinuation (Figure 1). A liver biopsy was performed, which confirmed cholestatic liver disease. Corticosteroids were initiated for immune modulation, and radioiodine therapy was planned as a definitive treatment for hyperthyroidism. Supportive care included continuation of cholestyramine for pruritus and strict avoidance of hepatotoxic medications. The patient remains under close monitoring, with follow‐up planned to evaluate his recovery and guide further management of his hyperthyroidism and liver dysfunction. This case highlights the rare occurrence of severe and prolonged carbimazole‐induced hepatotoxicity and underscores the importance of early recognition and comprehensive multidisciplinary management.

## 4 Conclusion and Results

Carbimazole‐induced hepatic injury is an extremely rare adverse event. However, this should be considered in patients presenting with jaundice and thyrotoxicosis. Prompt discontinuation of the drug and transition to other definitive treatments for hyperthyroidism is mandatory. Routine monitoring of liver function is crucial to detect and manage these rare toxic effects in patients receiving antithyroid medications. This unusual case report aimed to raise awareness among physicians about the potential for such severe complications associated with antithyroid drug use.

## 5 Discussion

Mechanism and Idiosyncrasy: Methimazole (carbimazole's active metabolite) similarly causes cholestasis, likely via immune‐mediated injury [5]. This reaction is idiosyncratic, not dose‐dependent, and typically occurs within weeks [1].

Drug‐induced liver injury is a significant clinical concern, with various mechanisms underlying its hepatotoxicity. The pathophysiology of DILI often involves an initial cellular insult, followed by mitochondrial permeability transition, and ultimately cell death. Clinically, DILI can manifest as acute or chronic conditions, including hepatitis, cholestasis, steatosis, and fibrosis [6].

Carbimazole is a commonly used antithyroid drug for thyrotoxicosis management. It is generally well tolerated, with reported side effects, including allergic skin reactions, gastrointestinal upset, and agranulocytosis. Although rare, liver injury is a serious adverse effect. Cholestatic jaundice without hepatic necrosis has been associated with carbimazole and its active metabolite methimazole, as observed during liver biopsy. Previous case studies have demonstrated intracanalicular cholestasis and minimal mononuclear cell infiltration in the portal triad, findings indicative of drug‐induced toxicity with no evidence of autoimmune or viral etiology [7].

In the present case, the patient developed signs and symptoms of hepatic injury 4 weeks after starting carbimazole therapy. He presented with progressive jaundice, severe pruritus, dark urine, and pale stool. On examination, he was jaundiced and exhibited fine tremors. Laboratory investigations revealed markedly elevated bilirubin (total bilirubin, 248 μmol/L; direct bilirubin, 216 μmol/L), elevated alkaline phosphatase (ALP, 366 U/L), and mildly elevated alanine aminotransferase (ALT, 54 U/L) and aspartate aminotransferase (AST, 35 U/L) levels. Extensive investigations, including hepatitis panel, HIV testing, and autoimmune markers (ANA, ANCA, AMA, and SMA), were negative. A review of the literature shows that the mean time of onset of jaundice after initiating thionamide therapy is approximately 36 days [5].

This variability in timing highlights the importance of regular monitoring of liver function throughout thionamide therapy. Healthcare providers should educate patients about the signs and symptoms of hepatotoxicity, emphasizing the need for prompt reporting of yellowing of the skin or eyes, dark urine, or abdominal pain.

Through the discussion about our patient and the literature review, ruling out thyrotoxicosis‐induced cholestatic liver injury requires thorough clinical correlation, exclusion of other causes, and an assessment of liver biochemistry. Clinically, symptoms such as fine tremors, weight loss, tachycardia, and heat intolerance, along with jaundice, may indicate thyrotoxicosis‐associated liver injury. Diagnosis is confirmed by elevated serum‐free T3 and free T4 with suppressed TSH levels. A detailed history is essential to rule out other potential causes, including alcohol use and drug‐induced liver injury—especially from antithyroid drugs such as methimazole or propylthiouracil—or herbal supplements. Laboratory investigations should include screening for viral hepatitis (HAV, HBV, and HCV), autoimmune liver diseases (ANA, ASMA, and LKM1 antibodies), and Wilson's disease (serum ceruloplasmin). Imaging studies, such as abdominal ultrasound or CT scan, help exclude biliary obstruction or hepatic lesions. Liver biochemistry typically shows elevated bilirubin and transaminases, with a mild‐to‐moderate pattern; a predominantly cholestatic pattern is rare but possible. If liver enzyme elevations or bilirubin levels are disproportionately high relative to thyroid dysfunction, alternative diagnoses should be considered. In terms of treatment, steroids such as prednisolone may be temporarily used to reduce systemic inflammation and suppress thyroid hormone release in severe cases, including thyroid storm or cholestasis. Improvement in liver function with steroid therapy may suggest an immune‐mediated or inflammatory component of thyrotoxicosis‐induced liver injury. However, definitive therapy for hyperthyroidism—through antithyroid medications, radioactive iodine, or surgery—remains the cornerstone of management, leading to the normalization of liver function over time. When hyperthyroidism is the primary cause of liver injury, liver function typically improves as thyroid hormone levels stabilize. The duration of steroid treatment is generally short term (1–2 weeks), with the exact course determined by clinical response, normalization of liver function, and initiation of definitive therapy. Prolonged steroid use is avoided unless clinically necessary due to potential adverse effects [8, 9, 10, 11, 12].

## Author Contributions

**Bassem Al Hariri:** supervision, validation, writing – original draft, writing – review and editing. **Mohammed Omer Elbadawi Elhassan:** writing – original draft, writing – review and editing. **Hadil Altaj Altrify Alsidig:** writing – original draft, writing – review and editing. **Muad Abdi Hassan:** supervision, writing – original draft, writing – review and editing. **Abdalrahman Mohammed Mostafa:** writing – original draft. **Memon Noor Illahi:** supervision, validation.

## Ethics Statement

This case report was subject to review and approval from the Institutional Review Board (IRB) of the Medical Research Center (MRC‐04‐24‐724) at Hamad Medical Corporation and is in full conformance with the principles of the “Declaration of Helsinki,” Good Clinical Practice (GCP), and within the laws and regulations of MoPH in Qatar. IRB of Hamad Medical Corporation waived the need for informed consent due to the retrospective nature of the study.

## Consent

Written informed consent was obtained from the patient for publication of this case report and any accompanying images.

## Conflicts of Interest

The authors declare no conflicts of interest.

## Data Availability

The data that support the findings of this study are available in this article. Further enquiries can be directed to the corresponding author.
